# Epidemiology of Toxoplasmosis among the Pakistani Population: A Systematic Review and Meta-Analysis

**DOI:** 10.3390/pathogens11060675

**Published:** 2022-06-10

**Authors:** Tehniat Shoukat, Usman Ayub Awan, Tahir Mahmood, Muhammad Sohail Afzal, Samia Wasif, Haroon Ahmed, Jianping Cao

**Affiliations:** 1 Department of Biosciences, COMSATS University Islamabad (CUI), Islamabad 45550, Pakistan; tehniatshoukat57@gmail.com; 2 Department of Medical Laboratory Technology, The University of Haripur, Haripur 22620, Pakistan; usman.ayub111@gmail.com; 3 Industrial and Systems Engineering Department, College of Computing and Mathematics, King Fahd University of Petroleum and Minerals, Dhahran 31261, Saudi Arabia; rana.tm.19@gmail.com; 4 Interdisciplinary Research Center for Smart Mobility & Logistics, King Fahd University of Petroleum and Minerals, Dhahran 31261, Saudi Arabia; 5 Department of Life Sciences, School of Science, University of Management and Technology (UMT), Lahore 54770, Pakistan; sohail.ncvi@gmail.com; 6 Department of Humanities, COMSATS University Islamabad (CUI), Islamabad 45550, Pakistan; samia.wasif@comsats.edu.pk; 7 National Institute of Parasitic Diseases, Chinese Center for Disease Control and Prevention, (Chinese Center for Tropical Diseases Research), Shanghai 200025, China; 8 Key Laboratory of Parasite and Vector Biology, National Health Commission of the People’s Republic of China, Shanghai 200025, China; 9 WHO Collaborating Center for Tropical Diseases, Shanghai 200025, China; 10 The School of Global Health, Chinese Center for Tropical Diseases Research, Shanghai Jiao Tong University School of Medicine, Shanghai 200025, China

**Keywords:** toxoplasmosis, humans, male, female, *Toxoplasma gondii*, prevalence, epidemiology, Pakistan

## Abstract

*Toxoplasma gondii* is an intracellular obligate parasite that causes toxoplasmosis, a zoonotic infection that affects warm-blooded animals and humans worldwide. To comprehensively characterize the disease condition in Pakistan for future reference, we ascertained the prevalence of *Toxoplasma* infection and predisposing factors in the Pakistani population over a 20-year period. We systematically reviewed research articles published in English (2000–2020) from PubMed and Google Scholar. The search results 26 publications involving 10,924 people and 2611 seropositive cases. The toxoplasmosis seropositivity rate was higher in women (25.44%) as compared to men (21.48%) and were statistically significant (*p* < 0.001). Furthermore, seropositivity was high among people with direct contact with cats, who consumed uncooked meat and raw vegetables, had poor education, and lived in rural areas. The 35–65-year age group had the highest prevalence rate of *T. gondii* infection. *Toxoplasma* infection was significantly more prevalent in Khyber Pakhtunkhwa province (25.87%) than in Punjab (20.42%) (*p* < 0.001). This is the first comprehensive analysis of *T. gondii* infection epidemiology in Pakistan. It reveals a high frequency of infection among women. We strongly encourage further research to aid patient care and the development of more efficient diagnostic tests and preventative techniques.

## 1. Introduction

*Toxoplasma gondii* (*T. gondii*), is a widely prevalent zoonotic infection affecting warm-blooded animals and humans globally [1,2]. *T. gondii* is transmitted to humans through contaminated water, raw vegetables containing the oocysts in cat feces, and undercooked and raw meat contaminated with tissue cysts [3] or vertically transmitted from the mother to the fetus. In addition, blood transfusions may also be reported as the potential route for transmission of *T. gondii* [4,5]. The intracellular obligate parasitic protozoa *T. gondii* affects many hosts [6]. Wild felines or cats are the definitive hosts of *T. gondii*, and as warm-blooded animals, humans are the intermediate host [7]. Other animals such as mice, goats, cattle, pigs, rats, and sheep also transmit toxoplasmosis [8,9,10]. Several factors are associated with *Toxoplasma* prevalence, including low socioeconomic status, gardening, nutritional habits, contact with domestic animals, and poor hygienic conditions [11]. In addition, geographic and climatic conditions also influence toxoplasmosis transmission [12]. Toxoplasmosis screening is critical for reproductive-age women, for whom testing identifies those at risk of infection and aids the management of innate toxoplasmosis [13].

The symptoms and signs of toxoplasmosis range from asymptomatic to life-threatening infections [6]. Several factors are associated with progress in toxoplasmosis, including *Toxoplasma* strain virulence and inoculum size, host immune system status, and genetic background [13]. The common symptoms include low fever, weakness, headache, myalgia, generalized lymphadenopathy, and serious defects such as chorioretinitis, pneumonia, and encephalitis [14]. Toxoplasmosis can lead to epilepsy, hydrocephaly, internal calcification, maternal death, mental retardation blindness, or spontaneous abortions during gestation [15]. Typically, toxoplasmosis is asymptomatic in infants at birth but later causes serious illness, ocular manifestation, headache, and other symptoms [16]. *T. gondii* leads to severe congenital disabilities such as hydrocephaly. In immunocompromised patients, toxoplasmosis presents symptoms such as apathy, seizures, confusion, visual disorder, dyspnea, personality changes, and diarrhea [17,18]. Up to 40% of patients with AIDS develop *T. gondii* encephalitis [3]. *T. gondii* is also a risk factor for personality changes, reduced intelligence, and schizophrenia [19].

*T. gondii* demonstrates cosmopolitan distribution worldwide [6,8,20]. At the global level, approximately 6 billion people are infected with *T. gondii*. Toxoplasmosis is prevalent in every country, and its seroprevalence in developing countries is quite high compared to that in developed countries. However, in developed and developing nations, the prevalence of toxoplasmosis ranges between 30 and 60% [21]. Its prevalence is 81% in Korea and 10.8% in the US [1]. The seroprevalence of *Toxoplasma* differs significantly among different age groups within the same area and different geographic regions within a country [22]. 

Few studies from Pakistan have reported that toxoplasmosis prevalence was 11.33–29.45% [9,23]. Furthermore, the seroprevalence of *T. gondii* in pregnant women varies; Punjab province reported the highest seroprevalence (63%), followed by Khyber Pakhtunkhwa (KPK, 38%) and Azad Jammu and Kashmir (AJK, 48%) [24]. With a total population of over 200 million people and a growth rate of approximately 2.04%, Pakistan is now ranked as the fifth most populated country globally [25,26]. On the other hand, Pakistan’s subtropical, tropical, humid, and rainy climate is changing, and environmental degradation, rising temperatures, ecological imbalance, changing the biodiversity, and other environmental variables affect the emergence of potential pathogens. However, there is a paucity of available studies and data, and toxoplasmosis prevalence in the human population of Pakistan remains uncertain. We conducted a systematic review and meta-analysis to ascertain the possible risk factors for *T. gondii* infection in the Pakistani population.

## 2. Results

The systematic review and meta-analysis evaluated 26 out of 1606 articles published from the years 2000 to 2020, as shown in Table 1. In total, the meta-analysis involved 10,924 people and 2611 seropositive cases (23.9%). All descriptive studies on prevalence and epidemiology of human toxoplasmosis in Pakistan were included.

The studies were conducted in two provinces: KPK (women = 7985; men = 2039) and Punjab (women = 900; men = 900). Toxoplasmosis prevalence was higher in women (25.44%) and relatively lower in men (21.48%), whereas the combined prevalence for both genders was 15.67% (cf. Table 2).

Toxoplasmosis seroprevalence was 6%, 19.07%, 25.82%, and 19.93% in the 25–40-, 35–65-, and >66-year age groups, respectively. The *T. gondii* infection prevalence rate was highest in the 35–65-year age group.

**Figure 1:**
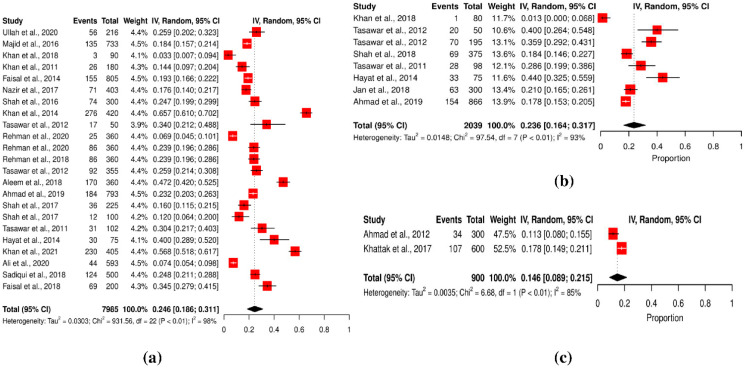
Forest schematic graph showing the prevalence of *T. gondii* infection in (**a**) females (**b**) males (**c**) both genders [3,9,15,22,24,27,28,29,30,31,32,33,34,36,37,38,39,40,41,42,43,44,45,46].

Gender-based association was statistically significant (*p* < 0.05). In contrast, the low prevalence rate in the combined female and male group (15.67%) was statistically significantly different (*p* < 0.0001). The meta-analysis demonstrated that the overall prevalence of toxoplasmosis in Pakistan was greater in the female population (25.44%) than in the male population (21.48%). In men and women combined, the association was statistically significant (women: Q statistic = 931.56, *p* < 0.001, tau^2^ = 0.0303, *I*^2^ = 97.6%; men: Q statistic = 97.54, *p* < 0.001, tau^2^ = 0.0148, *I*^2^ = 92.8%) (cf. Table 2; Figure 1a,b). However, thirty-three studies with a sample size of approximately 900 reported a prevalence of 15.6% for both genders combined (male and female) (Q statistic = 6.68, *p* < 0.001, tau^2^ = 0.0035, *I*^2^ = 85%) (cf. Table 2; Figure 1c).

**Figure 2:**
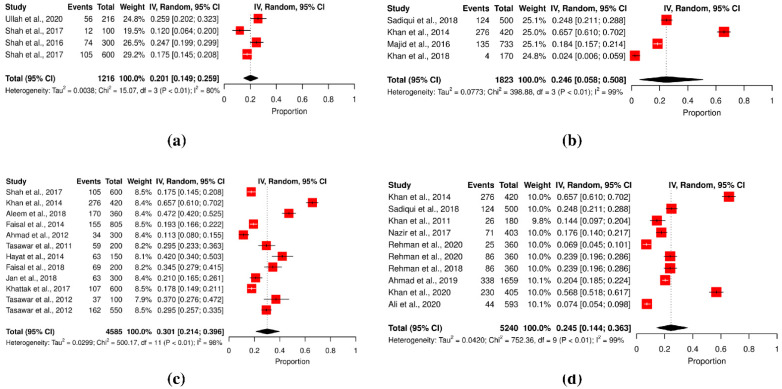
Forest schematic graph showing the different diagnostic techniques for detection of *T. gondii* infection. (**a**) LFCI, (**b**) other than LFCI, ELISA, and LAT (**c**) LAT (**d**) ELISA [3,9,15,22,23,24,27,29,30,31,32,33,36,37,38,39,40,41,42,43,44,45,46].

The included studies used a variety of serological diagnostic assays for determining anti-*T. gondii* IgG serum antibody levels. Enzyme-linked immunosorbent assay (ELISA, 24.92%, *n* = 5240) was the most used detection method, followed by the latex agglutination test (LAT, 28.35%, *n* = 4585), lateral flow chromatographic immunoassay (LFCI, 20.31%, *n* = 1216), immunochromatographic assay (ICA, 43.47%, *n* = 920), rapid diagnostic test (RDT, 2.35%, *n* = 170), and the indirect fluorescent antibody test (IFA, 18.41%, *n* = 733) (Q statistic = 15.07–752.32, *p* < 0.001, tau^2^ = 0.0038–0.0773, *I*^2^ = 80–99%) (cf. Table 2; Figure 2a–d).

**Figure 3:**
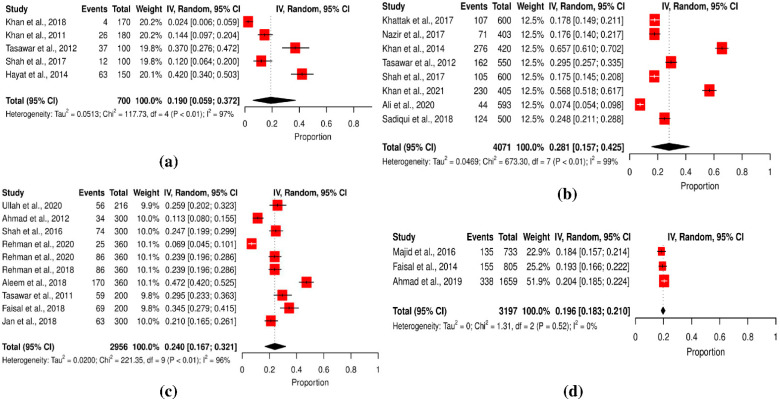
Forest schematic graph showing the studies of *T. gondii* infection with a sample size. (**a**) Less than 200, (**b**) in between 200–400, (**c**) between 400–600, (**d**) more than 600 [3,15,22,23,24,27,29,30,31,32,34,36,37,38,39,40,41,44,45,46].

Based on sample size, the *T. gondii* prevalence rate was higher in studies with sample sizes of 400–600 (27.47%), followed by studies with sample sizes of 200–400 (24.42%). In studies with sample sizes < 200, the incidence rate was 20.29%, and studies with sample sizes < 600 reported low prevalence (19.64%). These results were statistically significant (*p* < 0.0001) (Table 2). Ten studies involved 200–400 people, followed by studies that included 400–600 (*n* = 8), <200 (*n* = 5) and >600 people (*n* = 3) (Q statistic = 1.31–673.30, *p* < 0.001 and 0.51, tau^2^ = 0–0.0513, *I*^2^ = 0–99%) (cf. Table 2; Figure 3a–d).

**Figure 4:**
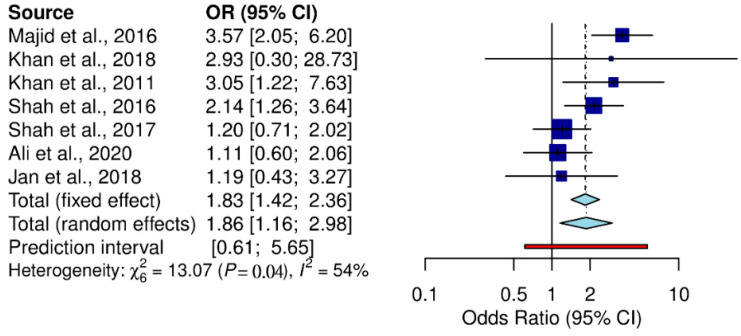
Forest schematic graph showing the studies of *T. gondii* infection in the rural and urban areas of Pakistan [3,31,32,36,37,42].

**Figure 5:**
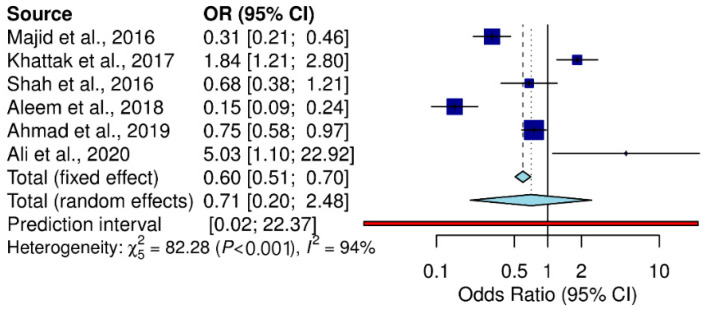
Forest schematic graph showing the studies of *T. gondii* infection among literate and illiterate peoples in Pakistan [3,15,31,32,33,39,43].

Residence was one of the main contributing factors in toxoplasmosis transmission. Our analysis demonstrated that rural areas reported the highest toxoplasma prevalence, followed by urban areas (Q statistic = 13.07, *p* = 0.04, tau^2^ = 0.1502, *I*^2^ = 54.1%) (cf. Table 2; Figure 4). The included studies divided educational background into literate and illiterate. Studies involving illiterate participants reported higher toxoplasma prevalence than studies with literate participants (Q statistic = 82.28, *p* < 0.001, tau^2^ = 1.3105, *I*^2^ = 93.9%) (cf. Table 2; Figure 5).

**Figure 6:**
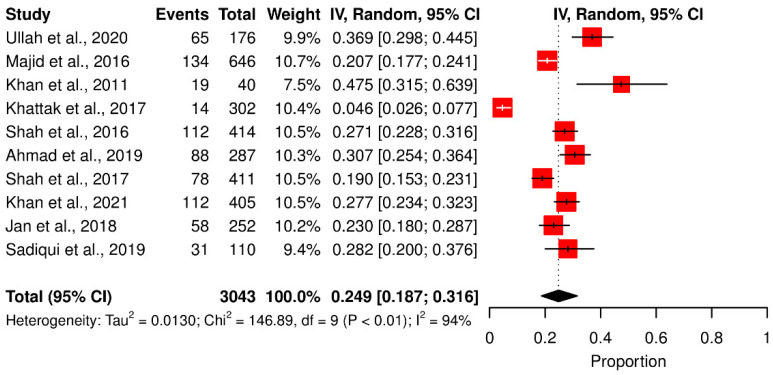
Forest schematic graph showing the studies of *T. gondii* infection among people who had contact with the animals [15,22,31,32,33,36,41,42,43,46].

**Figure 7:**
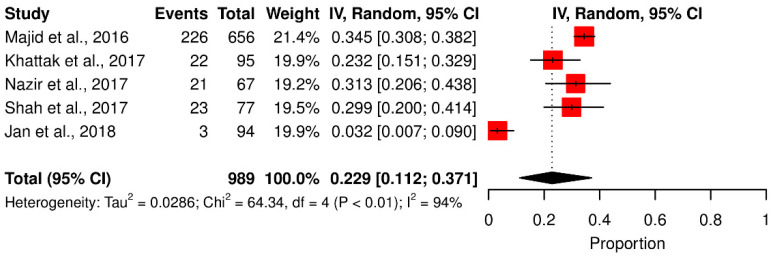
Forest schematic graph showing the studies of *T. gondii* infection among people who mostly eat undercooked food [31,33,34,36,42].

Subgroup analysis showed that toxoplasmosis prevalence was higher in the rural population (16.65%) than in the urban population (8.54%) but the difference was not significant (*p* > 0.005). Toxoplasmosis prevalence was higher in people who had direct contact with cats (Q statistic = 146.89, *p* < 0.001, tau^2^ = 0.0130, *I*^2^ = 93.9%) (cf. Table 2; Figure 6). Similarly, people who ingested undercooked meat and raw vegetables were more prone to contracting toxoplasmosis (Q statistic = 64.34, *p* < 0.001, tau^2^ = 0.0286, *I*^2^ = 93.8% (cf. Table 2; Figure 7).

**Figure 8:**
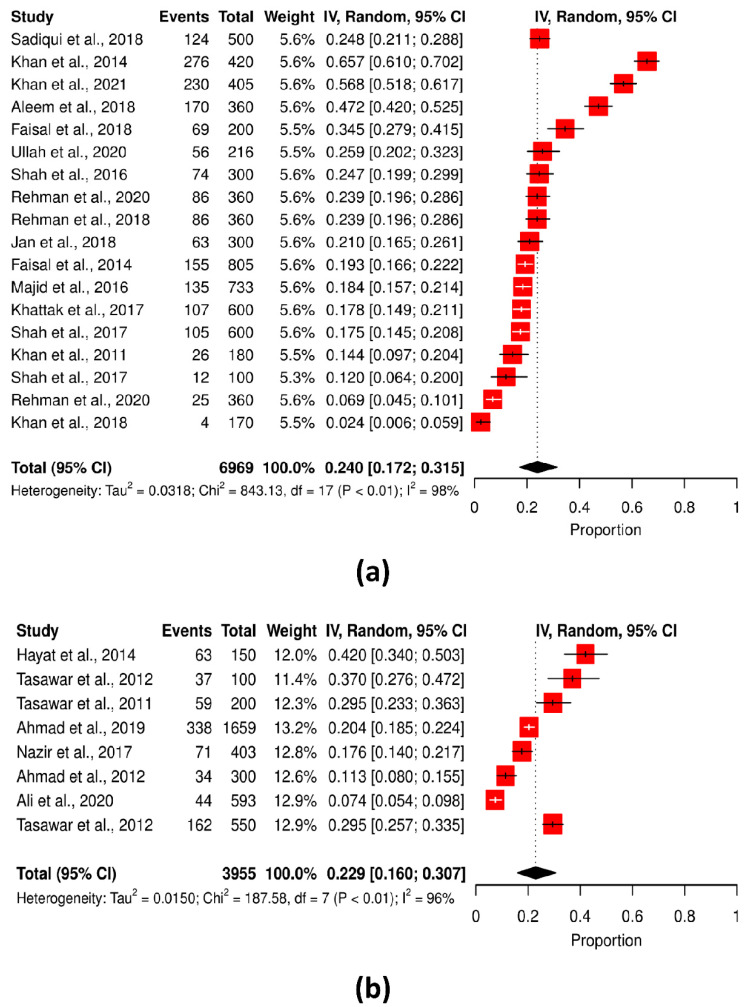
Forest schematic graph showing the studies of *T. gondii* infection in the (**a**) Khyber Pakhtunkhwa and (**b**) Punjab province, Pakistan [3,9,15,22,23,24,27,28,29,31,32,33,34,36,37,38,39,40,41,42,43,44,45].

**Figure 9:**
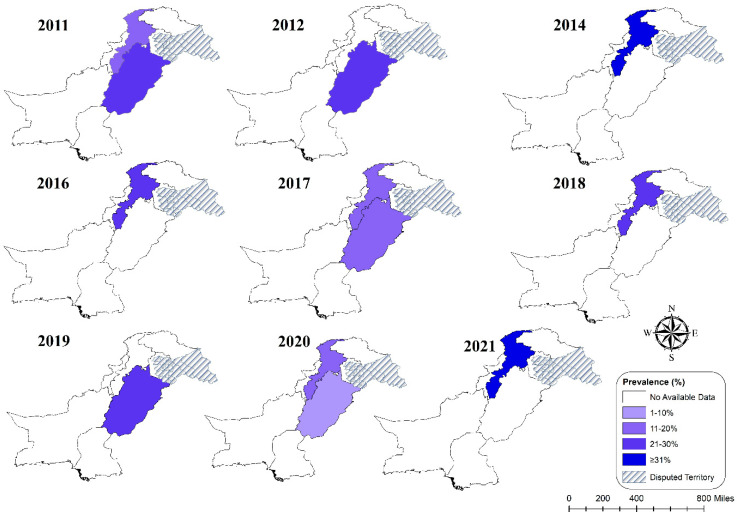
Spatial analysis of *T. gondii* infection prevalence report in different studies in different regions of Pakistan.

Higher *T. gondii* seropositivity was reported from KPK (25.87%), whereas Punjab reported the lowest prevalence (20.42%). The difference was statistically significant (Q statistic = 187.58–843.13, *p* < 0.001, tau2 = 0.015–0.0318, *I*^2^ = 0–99%) (cf. Table 2; Figure 8a,b). However, the overview of *T. gondii* prevalence was reported in various studies, as shown in Figure 9. Table 3 shows the seropositivity rate of *T. gondii* in the two provinces. Infection occurred more frequently in humid climates than in arid climates.

## 3. Discussion

It is critical to know how common *Toxoplasma* infection is among the general population of Pakistan. Using data from the literature obtained from different provinces, the present systematic review and meta-analysis assessed the epidemiology and prevalence of *T. gondii* infection in the general population of Pakistan. In a recent review, Rostami and colleagues summarized that the incidence rates of *T. gondii* infection among pregnant women were highest in the South American (56.2%) and African (48.7%) regions, while the lowest frequency was reported in the Western Pacific region (11.8%) [47]. Similarly, *T. gondii* seroprevalence was highest (75–85%) in Latin America, Central and Eastern Europe, and Southeast Asia [48]. Our meta-analysis findings reveal that approximately 24% of the general population of Pakistan was seropositive for the toxoplasmosis infection. Our findings support the widely held belief that approximately one-third of humanity is infected by *T. gondii* [49]. When it comes to the possibility of contracting toxoplasmosis, the most dangerous risk factors are eating raw or undercooked meat, having close contact with cats and soil, and engaging in inadequate hygiene standards [50,51,52]. Pakistan is classified as a country with a low-middle income; nevertheless, despite this, it is one of the top 10 nations in the world that does not have enough access to clean water. In addition, the lack of knowledge and the budgetary constraints in the health system in this nation all contribute to the spread of this infection [37,53], making the situation even more direr.

Epidemiological studies have been used for ascertaining *T. gondii* endemicity in humans and animals. There is a dearth of information on its seroprevalence [54,55]. Appropriate data on parasite prevalence and transmission routes would enable accurate risk assessment in pregnant women and aid the creation and implementation of parasitic infection control and preventive strategies [40,55]. Here, we determined that the total incidence and epidemiology of toxoplasmosis in Pakistan is higher in the female population (25.44%) than in the male population (21.48%), while combining male and female participants resulted in a low prevalence rate (15.67%) that was significantly different (*p* < 0.0001). Prevalence was predicted to be higher in women (25.44%), while the frequency was comparatively low in men (21.48%). In 2011, a study was performed in Kohat, KPK, to determine the seroprevalence and predisposing factors for *T. gondii* in pregnant women. Overall seropositivity was 14.4% (26 of 180) [15]. In 2016, a comparable study performed in the same zone obtained 733 random selections from prenatal facilities. Overall, seroprevalence was observed in 81.41% of pregnant women examined [31]. In a recent investigation, toxoplasmosis was prevalent in 24.36% of the male population [56]. Another study reported that the incidence rate of toxoplasmosis between 2000 and 2004 in the male population of the Czech Republic was 23% [57]. Our findings demonstrate that toxoplasmosis seroprevalence in the male population in Pakistan was 21.48%, which is in accordance with earlier research.

People with direct contact with cats, who ate undercooked meat and raw vegetables, and those with low levels of education (illiterate) had much higher *T. gondii* infection rates, with a statistically significant difference (*p* < 0.0001) between them (Table 2). A variety of risk factors have led to a high rate of toxoplasmosis. Reduced host immune responses, drinking polluted water and eating contaminated fruit and vegetables, owning pet cats, consuming undercooked meat, and coming into contact with the flesh or viscera of infected animals are all risk factors that increase toxoplasmosis occurrence [58,59]. Rostami et al. [47] have indicated a high incidence of congenital toxoplasmosis and acute *Toxoplasma* infection in people in low-income countries, and consuming raw or undercooked meat in the daily diet could be a significant predictor of toxoplasmosis. This is in line with our findings that people who directly touched cats, ate raw or undercooked meat and vegetables, or were illiterate had significantly higher *T. gondii* infection rates. *Toxoplasma* oocysts excreted in cat feces are more likely to survive and spread if the habitat contains atmospheric agents that aid their sporulation, such as sufficient humidity, which increases the risk of human and animal infection. As mentioned earlier, behavior is influenced indirectly by risk factors such as occupation, gender, geography, and educational attainment, influencing infection prevalence [59,60].

The subgroup analysis revealed higher toxoplasmosis prevalence in the rural population (16.65%) than in the urban population (8.54%), with no significant differences (*p* > 0.005). Cats and infected hunting animals are common in rural regions, as are higher oocyst survival rates in waste soil, a significant number of rural residents, inadequate health literacy, and restricted accessibility to health care facilities [60,61]. The subgroup analysis also revealed that prevalence estimates increased with age, consistent with previously reported data [47,58]. The data support our findings of inequalities among countries with different economic statuses. Overall, the total prevalence of toxoplasmosis in Pakistan is 23.9%. In contrast, the prevalence is lower in the US (10.8%), Mexico (20.26%), and China (6.31%) due to better hygiene practices, effective public awareness campaigns, accurate and consistent screening procedures for reproductive-age women and pregnant women, modernized livestock farming, well-established surveillance systems, and enhanced meat evaluation rules [4,62,63]. In addition, the likelihood of toxoplasmosis transmission by blood donation from healthy and asymptomatic carriers of infection adds to the worldwide prevalence, particularly in locations where toxoplasmosis is considerably more widespread [64]. People who test positive for *Toxoplasma* during the acute stage of the disease may have contributed to the expansion of toxoplasmosis through blood donation. Africa and Asia reported the highest and lowest global burdens of toxoplasmosis in blood donors at 46% (95% CI = 14–78) and 29% (95% CI = 23–35), respectively [65,66]. The highest toxoplasmosis seroprevalence was detected in Brazil (75%) and Ethiopia (73%) [65]. Pakistan is one of the top poultry producers in the world and chicken is the simplest source of meat, accounting for approximately 28% of high meat output. Increased chicken-eating appears to enhance the likelihood of *T. gondii* transmission to humans [67].

Here, toxoplasmosis seropositivity was 6%, 19.07%, 25.82%, and 19.93%, respectively, in the 25–40-, 35-, 35–65-, and >66-year age groups, respectively, indicating that *T. gondii* infection frequency is highest in the 35–65-year age group. According to research in Pakistan, infection was highest in the 26–40-year age cohort, accounting for 54.55% of the examined population, whereas the 41–55-year age group had low infection seropositivity, accounting for approximately 21.87%. The 15–25-year age group, on the other hand, had a moderate infection incidence of approximately 23.63%. Up to 76.1%, 45.1%, and 72.1% of infants in Tehsil Charsadda, Tangi, and Tehsil Shabqadar, respectively, were born with birth anomalies [40]. Similarly, another study from Pakistan reported that the 31–40-year age group had the highest toxoplasmosis incidence (33.33%, 30/110), followed by the 21–30-year age group (30%, 33/110) [46]. A similar pattern was reported in the US; while the frequency of toxoplasmosis may be declining, approximately 14% of people are seropositive by the age of 40 years [4].

The clinical manifestations of a *T. gondii* infection are not helpful in the diagnosis because they are not distinct [68]. The specific diagnosis of a *T. gondii* infection often relies on bioassays and serological testing, even though both methods have their limits in identifying or discriminating parasite subtypes [69,70]. The conventional toxoplasmosis diagnostic methods encompass etiological, immunologic, and imaging techniques. The advances in molecular technologies that can amplify parasite nucleic acids have led to significant advancements in the diagnostic process for toxoplasmosis. Among all these diagnostic methods, polymerase chain reaction (PCR) has the higher sensitivity and specificity in diagnosing the Toxoplasmosis [71,72]. In this meta-analysis, anti-*T. gondii* IgG serum antibody level was measured in different studies using a number of serological diagnostic tests. Enzyme-linked immunosorbent assay (ELISA, 24.92%, *n* = 5240) was the most used detection method, followed by the latex agglutination test (LAT, 28.35%, *n* = 4585), lateral flow chromatographic immunoassay (LFCI, 20.31%, *n* = 1216), immunochromatographic assay (ICA, 43.47%, *n* = 920), rapid diagnostic test (RDT, 2.35%, *n* = 170), and the indirect fluorescent antibody test (IFA, 18.41%, *n* = 733). 

Our subgroup analysis revealed that toxoplasmosis seropositivity frequency increased with advancing age, which is consistent with previously reported data [73]. Assessing the incidence of latent toxoplasmosis in different parts of the country can aid risk factor identification and the implementation of better policies and programs for reducing the disease burden. Therefore, we believe that our findings will aid future risk mapping and modeling. We also discovered regional differences and other parameters linked to the incidence of latent toxoplasmosis. Examining the underlying causes of this heterogeneity, particularly across locations or countries with similar features, would aid the development of better public health strategies. For example, if two countries had identical characteristics but vastly differing prevalence rates, lessons could be learned and implemented to reduce the burden of toxoplasmosis. Accordingly, a global approach to examining these variances and their consequences is required to inform action. Toxoplasmosis epidemiology varies geographically, requiring further epidemiological studies on socio-economic factors. These are most likely due to disparities in public health and hygiene facilities, socio-economic background, social and cultural conditions, and climate. Epidemiological research in Latin/Caribbean countries has revealed a high prevalence of *T. gondii* in feral cats, implying that the environment may be severely contaminated with oocysts, contaminating plants and people who touch the soil [74]. In contrast, this systematic review and meta-analysis have a number of limitations. For instance, although a complete search was done utilizing two databases, it is possible that some data were overlooked since certain findings were printed in local journals in languages rather than English; hence, local language articles were omitted.

To summarize, this systematic review and meta-analysis focused on the epidemiology and geographical location of *T. gondii* in Pakistan. Public understanding, training courses, and sanitary practice are all beneficial for guiding people toward cooked foods and cleaned vegetables, handling feral cats, and treating sick pet cats in a timely and proper manner. Intensive monitoring and routine inspections of food manufacturing businesses will enhance hygiene standards in abattoirs and the sanitary disposal of viscera, as would sophisticated screening programs utilizing modulus diagnostic tests, as shown in Table 1. Despite extensive research on *T. gondii* infection in humans, blood donors, pregnant women, immunocompromised patients, and animals (sheep, goats, cattle, and cats), there are no systematic reviews or meta-analyses on toxoplasmosis in ingested meats, or birds or other zoonotic hosts in Pakistan. In the future, we believe it would be beneficial for researchers and policymakers to concentrate their focus on the neglected populations.

## 4. Material and Methods

This was a systematic review for determining the prevalence of diseases caused by *T. gondii* in the Pakistani population (men and women). To reduce the potential for bias, data collection and eligibility criteria were designed for evaluating the crude prevalence (percentage of positive cases) and weighted population prevalence (proportion of positive cases) for every study. The frequency of infection over 20 years was depicted using forest plots.

### 4.1. Search Strategy

The search was limited to articles written in the English language. PubMed and Google Scholar were searched for papers published in 2000–2020 on toxoplasmosis infection prevalence, diagnostic tests, and risk factors for our systematic review and meta-analysis to determine *T. gondii* infection frequency in people in Pakistan. For data gathering, the English databases were searched using the following terms: “toxoplasmosis”, “*T. gondii*”, “humans”, “prevalence”, “epidemiology”, and “Pakistan” alone or in combination.

### 4.2. Data Collection

All 26 studies obtained were meticulously examined. The following information was extracted: year of publication, study demographic characteristics, author(s) name, study location, sample size, case numbers, diagnostic tests, and risk variables.

### 4.3. Inclusion and Exclusion Criteria

The inclusion criteria were established by abstract review, publication reading, and title screening. Duplicate papers, irrelevant data, inadequate data, and human-based research that did not focus on *T. gondii*-related disorders were excluded; studies conducted in Pakistan were retrieved.

### 4.4. Meta-Analysis

The meta-analysis was performed using R. First, *T. gondii* infection prevalence was determined using binomial distribution, $\hat{p}\hat{1}=\frac{x}{n}$ (1: Where *x* is the number of positive cases and *n* is the total number of examined cases) and the standard error $\left(se=\sqrt{\frac{\hat{p}\hat{q}}{n}}\right)$ for each study was computed. Next, the weighted prevalence of *T. gondii* was calculated for gender (female, male, and both), contact with animals, undercooked food, provinces (KPK and Punjab), and sample size (<200, 200–400, 400–600, and >600 people). For the above variables, we used a single-proportion meta-analysis. The Freeman–Tukey Double arcsine transformation was considered for the proportions and the inverse variances were estimated with the DerSimonian–Laird method. For the meta-analyses of location (rural and urban) and education (literate and illiterate), the odds ratios were calculated for the pooled effect size. Variance was estimated using the Sidik–Jonkman method and 0.1 increments were added for continuity correction of zero cells. The Cochran Q test and I^2^ statistic were used to determine between-study heterogeneity and forest plots (described as ES (effect size) with a 95% confidence interval (95% CI)) were constructed as a graphical summary of the results.

## 5. Conclusions

This review examines the current state of *T. gondii* infection in the Pakistani population. Toxoplasmosis was more common in women than in men. The high prevalence of *T. gondii* infection in Pakistan is a serious public health concern. Therefore, health measures should be taken to prevent and control the disease. Our findings provide important statistics and information on the prevalence of *T. gondii* infection, which could be helpful in infection management and control. In addition to serological techniques, more research is required to employ PCR tests to assess infection accurately. We also recommend multiple precautionary measures should be followed to control the risk factors of toxoplasmosis, including properly cooking meat and vegetables, using gloves while gardening, proper handwashing, and maintaining hygienic conditions. 

## Figures and Tables

**Table 1:** Baseline characteristics of studies included in the systematic review and meta-analysis.

Author	References	Year of Publication	Location	Diagnostic Method	Disease/Species Name	Sex	Total Individuals	Positive Cases	Prevalence (%)
Khan et al., 2011	[15]	2011	Kohat	ELISA	*T. gondii*	Female	180	26	14.40%
Tasawar et al., 2011	[27]	2011	Dera Ghazi Khan	LAT	*T. gondii*	Female	102	31	30.39%
		2011	Dera Ghazi Khan	LAT	*T. gondii*	Male	98	28	28.57%
Tasawar et al., 2012	[28]	2012	Rajanpur, Bahawalnagar, Shujabad, Multan	LAT	*T. gondii*	Female	355	92	25.90%
		2012	Rajanpur, Bahawalnagar, Shujabad, Multan	LAT	*T. gondii*	Male	195	70	35.89%
Tasawar et al., 2012	[23]	2012	Muzaffargarh	LAT	*T. gondii*	Female	50	17	34%
		2012	Muzaffargarh	LAT	*T. gondii*	Male	50	20	40%
Ahmad et al., 2012	[9]	2012	Lahore	LAT	*T. gondii*	Male and Female	300	34	11.30%
Khan et al., 2014	[24]	2014	Malakand Agency, Batkhela, Bahadur Khan, Rozi Khan Memorial Hospital	ICA, LAT, ELISA	*T. gondii*	Female	420	276	65.71%
Hayat et al., 2014	[29]	2014	Kallarwali, Muzaffar Garh	LAT	*T. gondii*	Female	75	30	40%
			Kallarwali, Muzaffar Garh	LAT	*T. gondii*	Male	75	33	44%
Faisal et al., 2014	[30]	2014	Swabi	LAT	*T. gondii*	Female	805	155	19.25%
Majid et al., 2016	[31]	2016	Upper Dir, Lower Dir, Swat	IFA Kit	*T. gondii*	Female	733	135	18.41%
Shah et al., 2016	[32]	2016	Chitral	LFCI	*T. gondii*	Female	300	74	24.70%
Khattak et al., 2017	[33]	2017	Mardan, Katlang, Takht Bhal	LAT	*T. gondii*	Female and Male	600	107	17.83%
Nazir et al., 2017	[34]	2017	Multan, Muzaffargarh, Dera Ghazi Khan, Layyah	ELISA	*T. gondii*	Female	403	71	17.60%
Shah et al., 2017	[35]	2017	Mardan	LFCI, LAT	*T. gondii*	Female	225	36	16.00%
			Mardan	LFCI, LAT	*T. gondii*	Male	375	69	18.40%
Shah et al., 2017	[36]	2017	Swabi	LFCI	*T. gondii*	Female	100	12	12%
Khan et al., 2018	[37]	2018	District Bannu	RDT	*T. gondii*	Female	90	3	1.77%
		2018	District Bannu	RDT	*T. gondii*	Male	80	1	0.59%
Rehman et al., 2018	[38]	2018	Mardan	ELISA	*T. gondii*	Female	360	86	23.89%
Aleem et al., 2018	[39]	2018	Matta, Upper Swat	LAT	*T. gondii*	Female	360	170	47.20%
Faisal et al., 2018	[40]	2018	Charsadda, Shabqadar, Tangi	LAT	*T. gondii*	Female and Male	200	69	34.50%
Sadiqui et al., 2018	[41]	2018	Mansehra, Hazara, Abbottabad	ICA, ELISA	*T. gondii*	Female	500	124	24.85%
Jan et al., 2018	[42]	2018	Charsadda, Shabqadar, Tangi	LAT	*T. gondii*	Male	300	63	21%
Ahmad et al., 2019	[43]	2019	Jhelum, Chakwal, Rawalpindi, Attock, Islamabad Capital Territory	ELISA	*T. gondii*	Female and Male	1659	338	20.37%
Ali et al., 2020	[3]	2020	Chiniot, Faisalabad, Jhang and Okara	ELISA	*T. gondii*	Female	593	44	7.42%
Rehman et al., 2020	[44] [45]	2020	Mardan	ELISA	*T. gondii*	Female	360	25	6.94%
		2020	Mardan	ELISA	*T. gondii*	Female	360	86	23.90%
Ullah et al., 2020	[46]	2020	Swat	LFCI	*T. gondii*	Female	216	26	26%
Khan et al., 2021	[22]	2021	Lower Dir	ELISA	*T. gondii*	Female	405	231	57.03%

ELISA = Enzyme-linked immunosorbent assay, LAT = latex agglutination test, RDT = rapid diagnostic test, LFCI = lateral flow chromatographic immunoassay, IFA = immunofluorescence assay, ICT = immunochromatographic assay.

**Table 2:** The estimated pooled effect size of the prevalence of toxoplasmosis infection in male and female population and detection methods in various regions of Pakistan through subgroup meta-analysis.

Type of Infection	Humans	No. of Studies	Sample Size	Positive Cases	Prevalence (%)	Cochran Q Statistic	*I*^2^ Statistic	*p*-Value †	Tau^2^
Toxoplasmosis	Female	23	7985	2032	25.44	931.56	97.6%	<0.0001	0.0303
	Male	08	2039	438	21.48	97.54	92.8%	<0.0001	0.0148
	Female and Male	02	900	141	15.67	6.68	85.0%	0.0098	0.0035
Detection method	LFCI	4	1216	247	20.31	15.07	80.1%	0.0018	0.0038
	ELISA	10	5240	1306	24.92	752.36	98.8%	<0.0001	0.0420
	LAT	12	4585	1300	28.35	500.17	97.8%	<0.0001	0.0299
	Others (ICA, RDT, IFA)	2	920	400	43.47	398.88	99.2%	<0.0001	0.0773
		1	170	4	2.35				
		1	733	135	18.41				
Sample size	<200	5	700	142	20.29	117.73	96.6%	<0.0001	0.0513
	200–400	10	2956	722	24.42	221.35	95.9%	<0.0001	0.0200
	400–600	8	4071	1119	27.47	673.30	99.0%	<0.0001	0.0469
	>600	3	3197	628	19.64	1.31	0.0%	0.5199	0
Residence *	Urban	7	1287	110	8.54	13.07	54.1%	0.0420	0.1502
	Rural	7	1585	264	16.65	13.07	54.1%	0.0420	0.1502
Education *	Literate	6	1935	355	18.34	82.28	93.9%	<0.0001	1.3105
	Illiterate	6	2043	449	21.97	82.28	93.9%	<0.0001	1.3105
Contact with animals	Female and Male	10	3043	711	23.36	146.89	93.9%	<0.0001	0.0130
Undercooked meat and raw vegetable consumption	Female and Male		989	295	29.82	64.34	93.8%	<0.0001	0.0286
Geographical region of Pakistan	KPK	18	6969	1803	25.87	843.13	98.0%	<0.0001	0.0318
	Punjab	8	3955	808	20.42	187.58	96.3%	<0.0001	0.0150

† *p*-value of heterogeneity statistic, * Education and Residence (Khan et al., 2021) were excluded due to incomplete data.

**Table 3:** Heterogeneity summary of prevalence according to province ^†^.

Province	No. of Studies	No. of People Examined	No. of Positive Cases	Prevalence (%)	Heterogeneity Test	
					*Q*	*P*
Khyber Pakhtunkhwa	18	6969	1803	25.87	843.13	<0.0001
Punjab	8	3955	808	20.42	187.58	<0.0001

^†^ Test of group difference.

## Data Availability

Not applicable.
